# Diagnostic efficacy of data mining method based on multimodal ultrasound for papillary thyroid carcinoma

**DOI:** 10.3389/fonc.2024.1439825

**Published:** 2024-10-24

**Authors:** Changyu Xu, Liwei Zhang, Qiming Zhang, Tianqi Wang, Yuqing Wu, Jinlai Yao, Xiaoqiu Dong

**Affiliations:** Department of Medical Ultrasound, Fourth Affiliated Hospital of Harbin Medical University, Harbin, Heilongjiang, China

**Keywords:** doppler ultrasound, elasticity imaging techniques, echography, papillary thyroid carcinoma, data mining

## Abstract

**Objective:**

The incidence of papillary thyroid caracinoma (PTC) is increasing year by year. Logistic regression model and Chi-squared automatic interaction (CHAID) decision tree based on multimodal ultrasound were established, and the diagnostic efficiency of the two models in PTC was compared.

**Methods:**

The findings, features and data of routine ultrasound, shear wave elastography (SWE) and contrast-enhanced ultrasonography (CEUS) were prospectively collected in 203 patients. Including: echogenicity, aspect ratio, maximum diameter of tumor, boundary, morphology, focal hyperecho, blood flow grading, maximum elasticity (E_max_), minimum elastcity (E_min_), mean elasticity (E_mean_), enhancement degree, enhanced characteristics, distribution of contrast agent, contrast medium arrival time. According to the pathological results, they were divided into PTC group and non-PTC group. CHAID decision tree model and binary Logistic regression model were established, receiver operator characteristic (ROC) curves of the two models were drawn, and diagnostic effectiveness was evaluated by comparing area under curve (AUC).

**Results:**

Logistic regression showed that hypoechoic or very hypoechoic, aspect ratio ≥1, microcalcification and high SWE value were risk factors for PTC (OR 8.604, 2.154, 2.297, 1.067, respectively, P < 0.05). The CHAID decision tree showed echo, aspect ratio, E_max_, contrast agent distribution and infusion time combined to diagnose PTC. ROC curve showed that the AUC of PTC predicted by Logistic regression model and CHAID decision tree model was 0.878 and 0.883, respectively, with no statistical significance (z=0.325, P=0.7456).

**Conclusion:**

Both Logistic regression model and CHAID decision tree model can play a good role in the diagnosis of PTC based on multi-modal ultrasound, but the diagnostic efficiency of both models is comparable. In conclusion, these two models provide new insights and ideas for PTC diagnosis.

## 1 Introduction

Thyroid nodules are a discrete lesion inside the thyroid gland and one of the most common diseases of the endocrine system. With the continuous improvement of imaging technology, the detection rate of thyroid nodules has gradually increased, and the prevalence rate in adults has reached as high as 60%, of which 3-4.2% of patients can be detected during physical examination alone (1). In patients with thyroid nodules, the incidence of thyroid cancer is approximately 1-5% (2). Meanwhile, thyroid cancer is the most common malignancy of the endocrine system. PTC is the most common pathological staging, with most PTC being only a few millimeters in diameter; PTC<1 cm in diameter are referred to as papillary thyroid microcarcinoma (PTMC). Although PTC has a good prognosis and is known as the “happy cancer”, 30%-40% of patients develop cervical lymph node metastasis (CLNM) (3). Because thyroid nodules are common in the population, we need a sensitive method to screen for malignant lesions. Due to the shallow location of the thyroid gland, high-frequency ultrasound is regarded as the preferred imaging technique for the diagnosis of thyroid-related diseases. It is the first non-invasive, convenient and quick method for the diagnosis of thyroid nodules, which can determine whether the lesions are located in the thyroid gland, and determine the shape, cyst consolidation, size, number, relationship between the nodules and peripheral tissues, and whether the cervical lymph nodes are enlarged (4).Owing to the superficial location of the thyroid gland, high-frequency ultrasound has become the imaging method of choice for diagnosing thyroid disorders, providing a noninvasive, convenient, and quick diagnostic method for the initial assessment of thyroid nodules. This technique was pioneered by Eleonora Horvath et al. in 2009, based on the Breast Imaging Reporting and Data System (BI-RADS) developed by the American College of Radiology, and applying concepts from breast ultrasound lesions to evaluate thyroid nodules; it was referred to as the Thyroid Imaging Reporting and Data System (TI-RADS) (5). Conventional ultrasound can diagnose benign and malignant nodules by comprehensively evaluating their boundaries, morphology, blood flow and internal echo, but studies have shown that about 30.8% of benign and malignant nodules have no significant difference in ultrasound images, resulting in an increased rate of missed diagnosis (6, 7). CDFI can measure the blood flow parameters of target lesions, thereby improving the accuracy of disease diagnosis. At present, CDFI has been widely used to evaluate the blood supply characteristics of thyroid nodules, so as to predict thyroid cancer. However, some scholars believe that CDFI has poor performance in detecting low-velocity blood flow, especially for low-velocity blood flow less than 1cm/s, which makes the evaluation of low-velocity blood flow have certain limitations. SWE is a technique that uses Young’s modulus to represent the velocity of shear waves generated and propagated by an ultrasonic transducer through an acoustic radiation force pulse within the tissue being tested, which can reflect the hardness of the tissue and be used to qualitatively and quantitatively assess the condition of the surrounding tissue. However, when nodular goiter is combined with thyroid cancer, there may be overlap in examining tissue hardness (8). A contrast-enhanced ultrasound (CEUS) CEUS or biopsy is required to determine the malignancy of the nodule, especially for nodules with an aspect ratio ≥1, microcalcifications, burr-like margins, or if the patient has a family history of thyroid cancer and significant radiation exposure (9). CEUS is a technique in which the microbubbles are injected into the vein, which produce nonlinear oscillation under the action of ultrasonic longitudinal waves, and the blood vessels of organs are visualized and quantified by real-time imaging software (10). However, CEUS requires intravenously injected contrast media and is a minimally invasive test. Although the description of focal microangiogenesis in CEUS may be more accurate than the changes that can be evaluated by Doppler ultrasound, the characteristics of benign and malignant thyroid nodules in CEUS are still at the research level, and the current diagnostic criteria need to be further explored (11). Fine needle aspiration biopsy (FNAB) under ultrasound guidance is the “gold standard” for the minimally invasive diagnosis of papillary thyroid cancer. However, “false negative” pathology results may be obtained if the necrotic component of the cancer tissue is collected, or if a lesion is too small to obtain effective tissue for analysis. The procedure relies on the physician’s technical level. Therefore, a model that can combine multimodal ultrasound to diagnose papillary thyroid cancer can improve diagnostic efficiency, reduce trauma, and prevent overdiagnosis. Due to the advantages and limitations of different new ultrasound technologies, the combined diagnosis of thyroid nodules with TI-RADS guidelines established on the basis of conventional gray scale ultrasound and gray scale ultrasound can improve the diagnostic efficiency of thyroid nodules.

Decision tree model and Logistic regression model are common data mining methods. Because of its simple preprocessing, easy integrated learning and good fitting ability, decision tree model is widely used in the extraction of various imaging features, the differentiation of similar diseases and the evaluation of clinical treatment effect. Logistic regression model is widely used in clinical research in various fields, especially in epidemiological analysis, which is commonly used in automatic diagnosis of diseases, exploration of risk factors causing diseases and prediction of the possibility of disease occurrence. These two models can improve the quantity and quality of knowledge stored in data mining models, and can extract the similarities of similar cases and the differences between different classes (12), becoming representatives of multivariate functions that can be used in daily life. At present, there are many researches using CHAID decision tree model or binary Logistic regression model alone, but there are few literatures on which model is suitable for PTC diagnosis. In this study, various ultrasonic features of clinical PTC collected were used to establish CHAID decision tree and binary Logistic regression model, and a better diagnostic model was selected to provide help for clinical treatment.

## 2 Methods

### 2.1 Study object

A total of 253 patients with 253 thyroid nodules were prospectively collected from the Fourth Affiliated Hospital of Harbin Medical University from December 2021 to October 2023. (If the patient underwent surgery after puncture, the postoperative pathological results were selected; If a patient can detect multiple nodules, the one with the most malignant signs under ultrasound characteristics will be selected), and the one with unclear pathological results will be added immunohistochemistry. Inclusion criteria: ① Complete ultrasonic examination data (including conventional ultrasound, SWE and CEUS); ② The pathological results were accurate; ③ Never experienced radiation or chemotherapy or other related medical treatment; ④ Age 18-80 years old; ⑤ No other malignant tumor disease or metastasis. Exclusion criteria: ① the ultrasonic examination data is incomplete; ② Non-biopsy or non-surgical patients; ③ Patients with thyroid disease affecting the observed nodules; ④ Patients with recurrence; ⑤ The pathology was other malignant lesions except PTC. After exclusion, 203 subjects with 203 nodules were included. According to the pathological results, 111 cases were divided into PTC group and 92 cases were non-PTC group (119 cases were pathological after operation and 84 cases were pathological after puncture; There were 36 nodular goiter, 18 follicular tumors, 4 adenomas, 7 chronic lymphocytic thyroiditis, 26 papilloma, and 1 subacute granulomatous thyroiditis). The study has been formally approved by the Ethics Committee and all participating patients have signed informed consent forms (Reference number: 2021-WZYSLLSC-26).

### 2.2 Instruments and methods

#### 2.2.1 Instruments

Routine ultrasound and elastography were performed using a HOLOGIC Aixplorer ultrasound scanner equipped with an SL15-4 line-array probe (probe frequency 4-15 MHz); CEUS was performed using a Logiq E20 ultrasound scanner equipped with an L2-9 probe (probe frequency 2-9 MHz); and ultrasound guided puncture biopsy was performed using a Logiq E20 ultrasound scanner equipped with an ML6-15 probe (probe frequency 6-15 MHz).

#### 2.2.2 Conventional ultrasound

**Figure 1:**
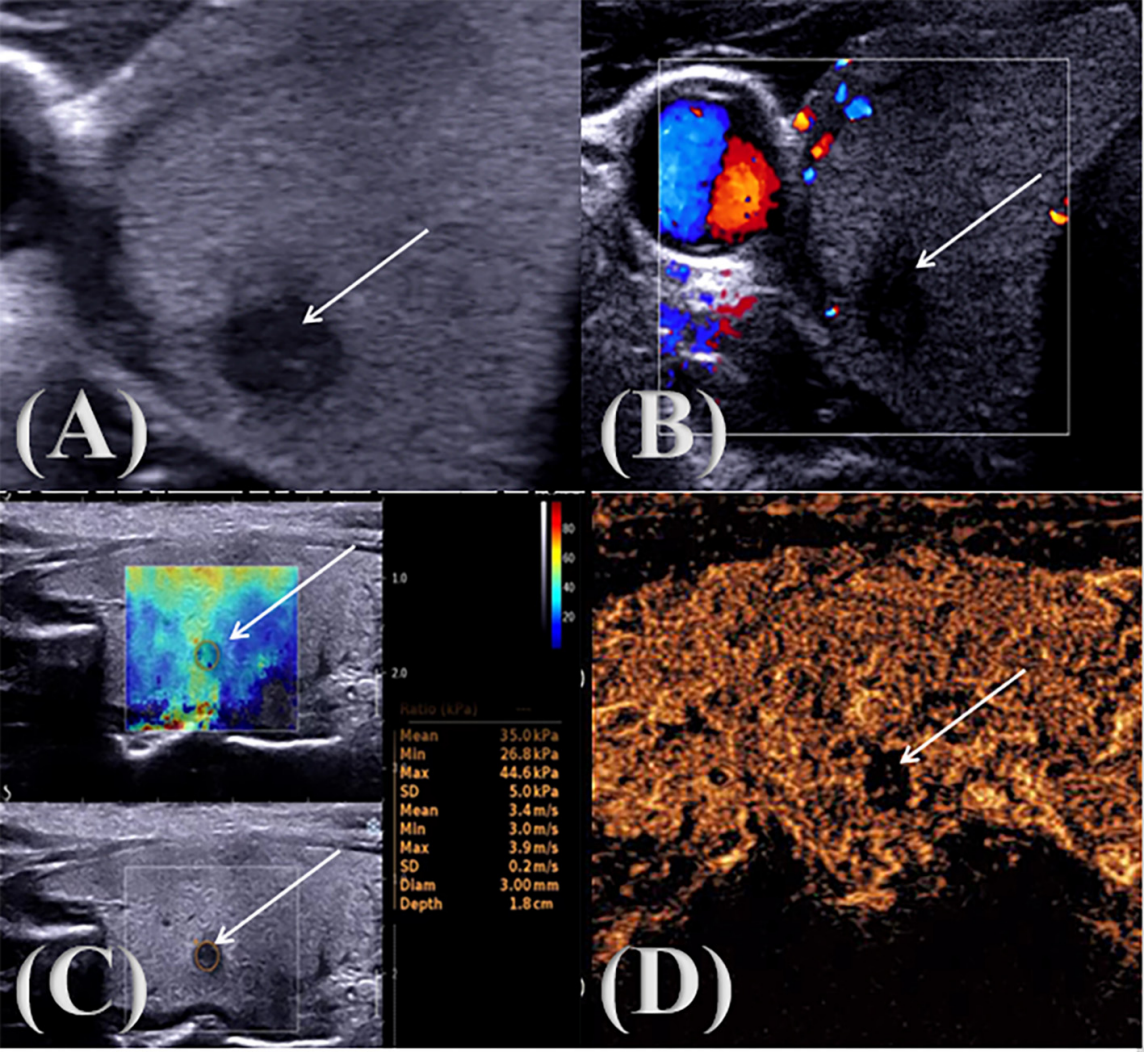
PTC multimodal ultrasound image. ↑: Lesion **(A)** two-dimensional gray scale ultrasonic image; **(B)** CDFI audio image; **(C)** SWE image; **(D)** CEUS audio image.

**Figure 2:**
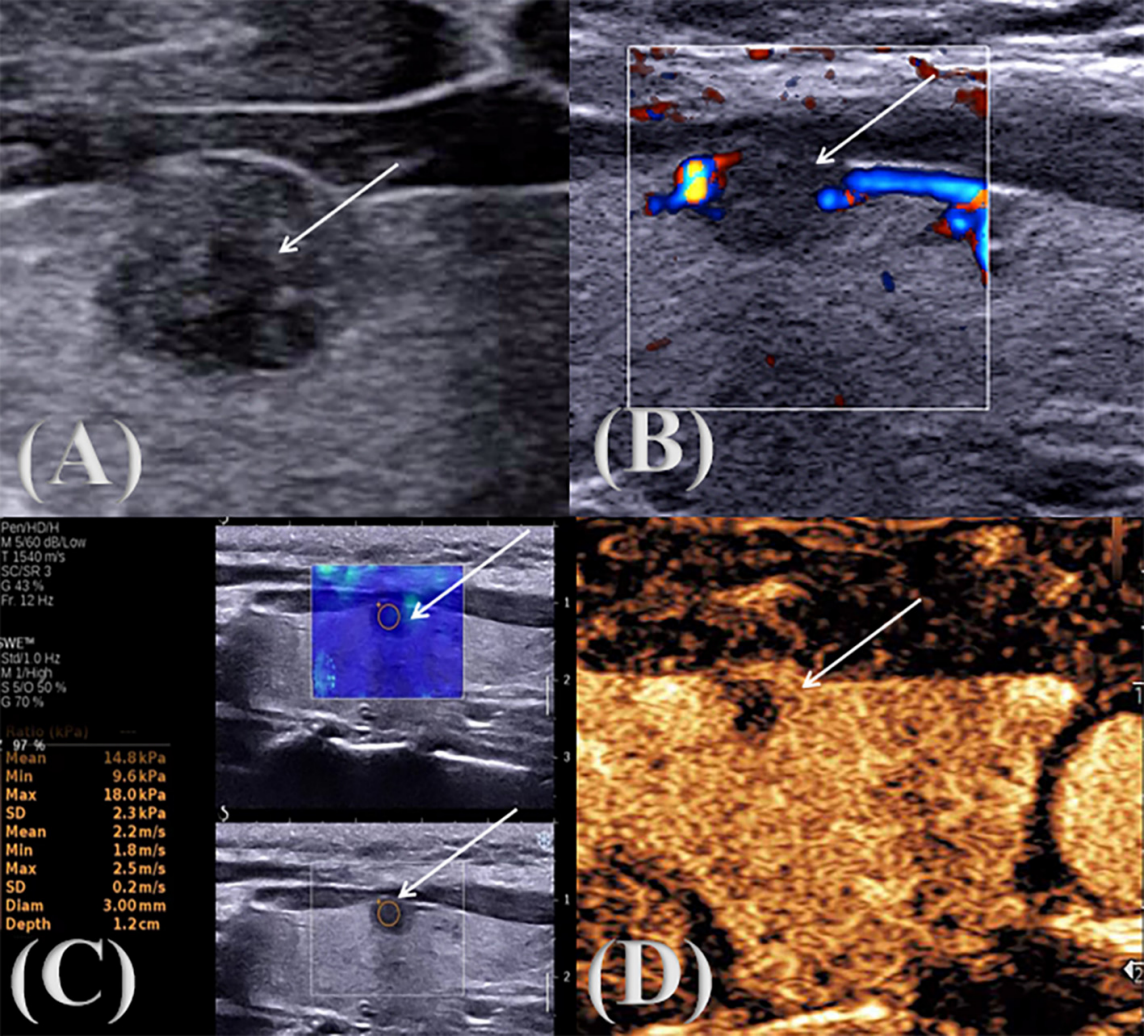
Multimodal ultrasound image of a pathologically confirmed nodular goiter. ↑: Lesion **(A)** two-dimensional gray scale ultrasonic image; **(B)** CDFI audio image; **(C)** SWE image; **(D)** CEUS audio image.

Conventional ultrasonography was shown in **Figures 1A, B**, **2A, B**. The patient was positioned supine on a diagnostic bed, with the head tilted upward to fully expose the neck. The bilateral lobes and isthmus of the thyroid gland were fully scanned, and the nodules were graded according to the TI-RADS criteria developed by the American College of Radiology. The maximum diameter (≤1 cm, >1cm); aspect ratio (<1, ≥1); echogenicity (isoechoic or hyperechoic, hypoechoic or very hypoechoic, mixed cystic and solid echogenicity), boundary (clear, unclear); morphology (regular, irregular or lobulated, invasion of surrounding thyroid tissue); focal strong echo (no strong echogenicity, punctate strong echogenic light spots, gross calcification or peripheral calcification are seen within the lesion); blood flow grading was classified into Grades 0 to III (Grade 0: no blood flow signal in the lesion; Grade I: 1~2 punctate blood flow signals or fine-pointed rod-shaped blood flow signals in the nodule, i.e., few blood flow signals; Grade II: 3~4 punctate blood flow signals in the nodule or a blood vessel whose length is close to or exceeds the radius of the lesion, i.e., more blood flow; Grade III: 5 or more punctate blood flow signals or 2 or more punctate blood flow signals in the nodule, i.e., rich blood flow (13); focal strong echo (no strong echogenicity, punctate strong echogenic light spots, gross calcification or peripheral calcification are seen within the lesion). The examination was performed by two physicians with more than 10 years of experience in ultrasound diagnosis. When the diagnosis was different, the two doctors discussed it together and reached a conclusion.

#### 2.2.3 SWE

During the examination, a suitable sampling frame was selected according to the size of the lesion, and the patient was asked to keep the head tilted back, hold the breath and keep the image stable for 3s, and the sample frame was placed on the hardest part of the lesion, frozen, and the E_max_, E_min_, E_mean_ were measured and stored in the image, as shown in **Figures 1C**, **2C**. The examination was conducted by two physicians with more than 10 years of experience in ultrasound diagnosis. Each of them measured three times, a total of 6 times, and took the average value respectively. Finally, the Emax, Emin, and Emean of the nodules were obtained and the results were recorded.

#### 2.2.4 CEUS

Ultrasonography was shown in **Figures 1D**, **2D**. The patient was placed in the supine position, and the optimal blood-supplying section of the target contrast lesion was confirmed under routine ultrasonography. The probe was stabilized and the contrast mode was set 2.4 ml of contrast agent (sonovol, Bracco, Italy) was injected through the cubital vein, and 5 ml of saline was rapidly injected. CEUS images were observed dynamically for 3 min, and the degree of enhancement of the nodule (hypointensification, isointensification, hyperintensification), the characteristics of the enhancement (irregular enhancement, centripetal enhancement, circumferential enhancement), the distribution of the contrast agent (homogeneous, inhomogeneous), and contrast medium arrival time (earlier than the adjacent thyroid tissue, synchronized with the adjacent thyroid tissue, later than adjacent thyroid tissue) were recorded (14). The examination was performed by two physicians with more than 10 years of experience in ultrasound diagnosis. When the diagnosis was different, the two doctors discussed it together and reached a conclusion.

#### 2.2.5 Ultrasound-guided thyroid nodule aspiration biopsy or surgery

The patient was placed in a supine position, the puncture site was selected under ultrasound guidance, the anterior neck skin is disinfected, and 1% lidocaine 5 ml is used for local anesthesia. The entry point was selected under ultrasound guidance, and the 22G biopsy needle was inserted into the target thyroid nodule. If there was a suspicious nodule, the suspicious nodule was punctured; if suspicious nodules were found on both sides, both sides were punctured; if no suspicious nodule was found, the largest solid or solid predominant nodule was punctured. The biopsy needle was quickly lifted several times in the nodule at multiple angles and the needle was withdrawn. Two smears were collected, and the operation was repeated two more times for a total of six smears. The needle was withdrawn, pressure was applied to stop bleeding, and a dressing was applied. The procedures were all performed by the general surgery department of the Fourth Affiliated Hospital of Harbin Medical University.

### 2.3 Statistical methods

SPSS 25.0 software was used for statistical processing. The Shapiro-Wilk (SW) method was used to check whether the measurement data fit the normal distribution. For measurement data conforming to the normal distribution, the mean ± standard deviation ( $\bar{\text{x}}$ ± s) was used, and the independent sample T-test was used to assess the differences between the groups. Measurement data that did not conform to the normal distribution were represented by the quartile [M (P25 P75)], and the difference between the two groups was compared by Mann-Whitney U test. The Receiver operator characteristic (ROC) curves of the three kinds of elastic imaging results were drawn, and the elastic value with the best diagnostic efficiency was selected by comparing the Area under the curve (AUC). n (%) was used to represent the count data, and univariate analysis was performed using the χ^2^ test. Multivariate analysis was performed before Logistic regression modeling. Features with statistically significant differences (P < 0.05) were selected as independent variable X, and ultrasonic features without statistical differences were excluded. The pathological results were the dependent variable Y. A binary Logistic regression model was established, and the assignment was shown in **Table 1**. Independent variables with independent prediction ability of malignant lesions were screened out (P < 0.05), and their OR values were calculated, and then the risk degree was compared. CHAID DT algorithm was used to construct the PTC prediction model, and the DT model was tested by the ten-fold cross-validation method. The ROC curves of the two models were drawn respectively, and the AUC of the two models were compared, so as to compare the PTC prediction efficiency of the two models. All AUC differences were compared using MedCalc software.

**Table 1:** Various characteristics of thyroid nodules.

Nodule characteristics	Variable names	Description of assignments
Gender	X_1_	male=0, female=1
Maximum diameter	X_2_	≤1cm=0, >1cm=1
Boundary	X_3_	clear=0, unclear=1
Morphometric	X_4_	regular=0, Irregular or lobulated or invade surrounding thyroid tissue =1
Echogenicity	X_5_	Isoechoic or hyperechoic or mixed cystic and solid echogenicity=0, hypoechoic or very hypoechoic =1
Aspect ratio	X_6_	<1 = 0, ≥1 = 1
Blood flow grading	X_7_	Grade 0 or Grade I=0, Grade II or Grade III =1
Focal strong echo	X_8_	absent or gross calcification or marginal annular calcification =0, dotted strong echoes =1
Degree of enhancement	X_9_	isointensification or hyperintensification=0, hypointensification=1
Characteristics of enhancement	X_10_	irregular or circumferential enhancement =0, centripetal enhancement =1
Distribution of contrast agent	X_11_	Homogeneous =0, inhomogeneous =1
Contrast agent arrival time	X_12_	earlier than adjacent thyroid tissue or synchronized =0, later than adjacent thyroid tissue =1
E_max_	X_13_	
E_min_	X_14_	
E_mean_	X_15_	
Age	X_16_	

## 3 Results

### 3.1 General information

A total of 203 thyroid nodules were collected from 203 patients, including 111 with PTC and 92 without PTC (36 with nodular goiter, 18 with follicular tumor, 4 with adenoma, 7 with chronic lymphocytic thyroiditis, 26 with papilloma, and 1 with subacute granulomatous thyroiditis). All patients were 18~79 years old, and the ages of PTC group and non-PTC group were (40.95 ± 10.65) years old and (44.74 ± 10.71) years old, respectively, with no statistical significance (P=0.418). The maximum length of nodules was 0.2~8.8cm, which was 0.70 (0.50, 1.10) cm and 0.82 (0.53, 1.34) cm in the two groups, respectively, and the difference between them was statistically significant (P=0.035). The E_max_ of the two groups was 53.10 (42.92, 76.35) kPa and 30.20 (23.08, 40.15) kPa, and the E_min_ was 34.45 (24.55, 49.43) kPa and 15.55 (9.95, 22.93) kPa, respectively. E_mean_ values were 49.35 (37.38, 67.16) and 24.80 (18.65, 33.90) kPa, respectively, with statistical significance (P < 0.001), as shown in **Table 2**. The ROC curves of E_max_, E_min_ and E_mean_ were plotted, and the AUC was 0.842, 0.829 and 0.840, respectively, but there was no statistical significance among them (P > 0.05). We selected E_max_ with relatively maximum AUC for subsequent modeling, as shown in **Table 3**. The benign and malignant critical value of E_max_ was 41.25kPa, the sensitivity was 78.4%, the specificity was 80.4%, and the Yoden index was 0.588.

**Table 2:** Comparison of results of the measures in the PTC and non-PTC groups (individuals).

Groups	PTC	non-PTC	Z(t)	P
Number of nodules (n)	111	92		
Age (x ± s, year)	40.95 ± 10.65	44.74 ± 10.71	-2.514^a^	0.418
Maximum diameter[M(P25,P75),cm]	0.70 (0.50,1.10)	0.82 (0.53,1.34)	-2.106	0.035
E_max_[M(P25,P75),kPa]	53.10 (42.92,76.35)	30.20 (23.08,40.15)	-8.394	0.001
E_min_[M(P25,P75),kPa]	34.45 (24.55,49.43)	15.55 (9.95,23.39)	-8.068	0.001
E_mean_[M(P25,P75),kPa]	49.35 (37.38,67.18)	24.80 (18.65,33.90)	-8.337	0.001

^a^ denotes t value.

**Table 3:** Comparison of the region between the E_max_, E_min_ and E_mean_ curves.

Variables	AUC	Std.Error	*P*	95%CI
E_max_	0.842	0.029	0.001	0.787-0.898
E_min_	0.829	0.029	0.001	0.772-0.886
E_mean_	0.840	0.029	0.001	0.784-0.896

### 3.2 Characteristics analysis of PTC and non-PTC nodules

There were no statistically significant differences in nodule gender, maximum diameter, boundary, blood flow grading, enhancement degree, enhancement characteristics, and contrast agent infusion time between PTC group and non-PTC group (P > 0.05), but there were statistically significant differences in nodule morphology, echo, aspect ratio, intra-nodule calcification and E_max_ (P < 0.05). Most nodules in PTC group were characterized by low or extremely low echo (97.3%), maximum diameter ≤1cm, aspect ratio ≥1, irregular or lobed shape or invasion of the surrounding thyroid tissue, punctiform strong echo visible in nodules, and uneven contrast agent perfusion during angiography. In the non-PTC group, nodules with regular shape, aspect ratio < 1, no obvious calcification and uniform centripetal perfusion were found, as shown in **Tables 4**, **5**.

**Table 4:** Evaluating the independent factors of gender and the count of standard ultrasound features between the PTC group and the non-PTC group n (%).

Characteristics	PTC (n=111)	non-PTC (n=92)	χ^2^	P
Gender				
male	22 (19.8)	16 (17.4)	0.195	0.659
female	89 (80.2)	76 (82.6)		
Maximum diameter				
≤1cm	81 (73.0)	57 (62.0)	2.805	0.094
>1cm	30 (27.0)	35 (38.0)		
Boundary				
clear	45 (40.5)	45 (48.9)	1.429	0.232
unclear	66 (59.5)	47 (51.1)		
Morphology				
regular	35 (31.5)	52 (56.5)	12.828	0.001
irregular or lobulated or invade surrounding thyroid tissue	76 (68.5)	40 (43.3)		
Echogenicity				
isoechoic or hyperechoic	2 (1.8)	4 (4.3)	36.633^a^	0.001^a^
mixed cystic-solid echo	1 (0.9)	25 (27.2)		
hypoechoic	108 (97.3)	63 (68.5)		
Aspect ratio				
<1	38 (34.2)	59 (64.1)	18.020	0.001
≥1	73 (65.8)	33 (35.9)		
Focal strong echo				
no strong echo	37 (33.3)	60 (65.2)	20.120	0.001
dotted strong echo	56 (50.5)	22 (23.9)		
gross calcification or peripheral calcification	8 (7.2)	10 (10.9)		
Blood flow grading				
Grade 0	92 (82.9)	66 (71.7)	4.037	0.256
Grade I	8 (7.2)	13 (14.1)		
Grade II	8 (8.1)	10 (10.9)		
Grade III	21 (1.8)	3 (3.3)		

a indicates that the Fisher’s exact probability method was used.

**Table 5:** Comparison of CEUS feature count independent variables between PTC group and non-PTC group n (%).

Characteristics	PTC (n=111)	non-PTC (n=92)	χ^2^	P
Degree of enhancement				
hypointensification	66 (59.5)	42 (45.7)	5.476	0.065
isointensification	23 (20.7)	32 (34.8)		
hyperintensification	22 (19.8)	18 (19.5)		
Enhancement characteristics				
centripetal	56 (50.5)	40 (43.5)	1.522	0.467
irregular	25 (22.5)	20 (21.7)		
circumferential	30 (27.0)	32 (34.8)		
Distribution of contrast agent				
homogeneous	84 (75.7)	29 (31.5)	39.740	0,001
inhomogeneous	27 (24.3)	63 (68.5)		
Contrast agent arrival time				
earlier than	23 (20.7)	19 (20.7)	1.852	0.396
synchronized	34 (30.6)	36 (39.1)		
later than	54 (48.7)	37 (40.2)		

### 3.3 Logistic regression model based on multi-mode ultrasonic PTC was established

Independent variables with statistically significant differences, such as nodule echo, aspect ratio, shape, calcification and E_max_, were included in the binary Logistic regression model of ultrasonic signs, and the corresponding regression formula Logit (P) =-5.166 + 2.152X_5_+0.767X_6_+0.831X_8_+0.065X_13_ was constructed. The OR values of the respective variables in the model from high to low were as follows: echo (X_5_) > calcification situation (X_8_) > aspect ratio (X_4_) > E_max_ (X_13_). This model was used to predict 203 nodules in this study. Regression value > 0.5 was defined as PTC, ≤0.5 was defined as non-PTC, and the prediction accuracy was 81.3%, as shown in **Table 6**.

**Table 6:** PTC binary Logistic regression model.

Ultrasonic characterization	Partial regression coefficient	Standard error	Wald χ^2^	P	*OR *(95%CI)
Echo (X_5_)	2.152	0.696	9.574	0.002	8.604 (2.201~33.633)
Aspect ratio (X_6_)	0.767	0.377	4.153	0.042	2.154 (1.030~4.505)
Calcification (X_8_)	0.831	0.379	4.804	0.028	2.297 (1.092~4.831)
E_max_(X_13_)	0.065	0.012	30.414	0.001	1.067(1.043~1.092)
Constant term	-5.166	0.844	37.466	0.001	0.006(-)

### 3.4 Establish PTC CHAID DT prediction model based on multi-mode ultrasound

**Figure 3:**
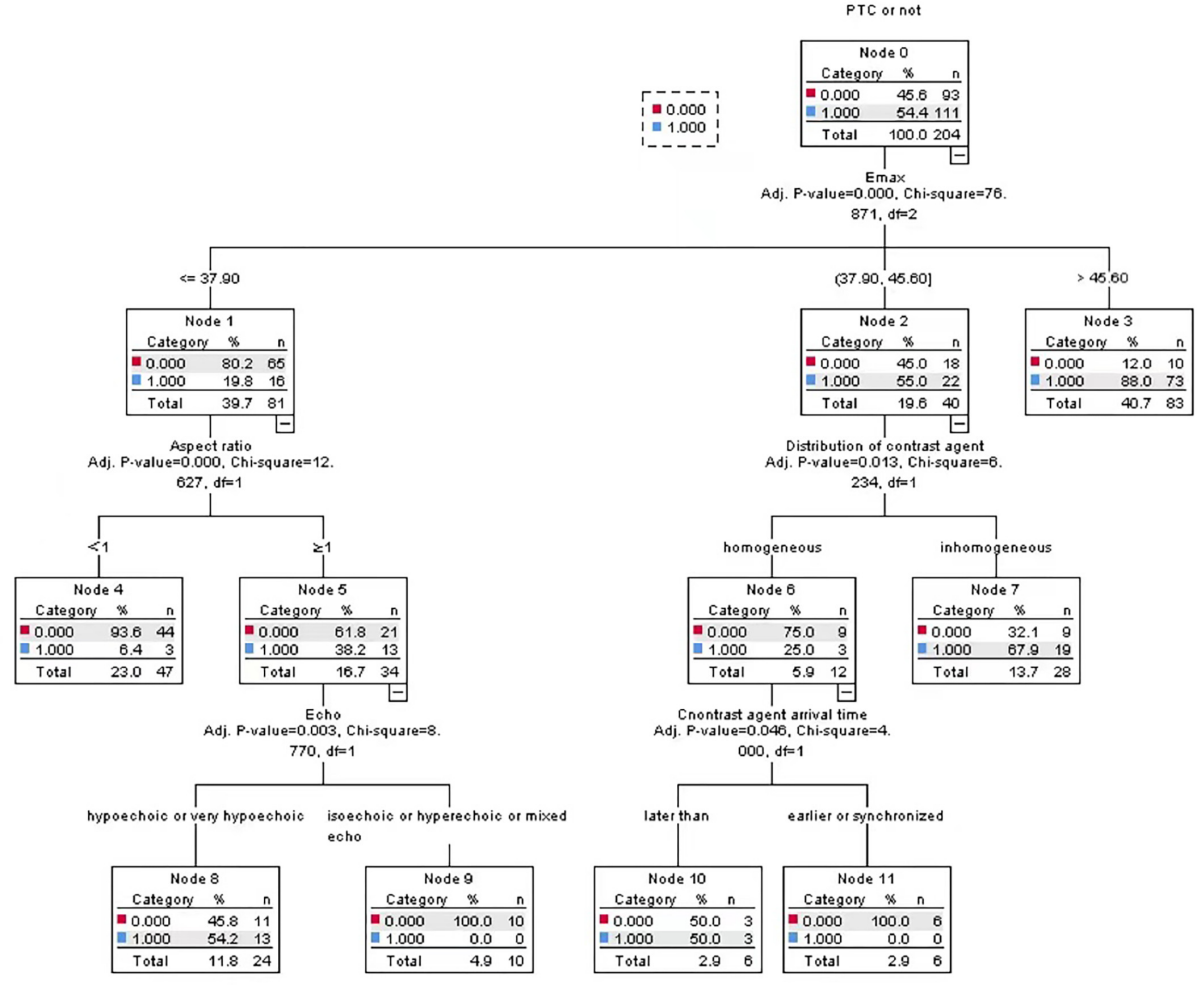
CHAID DT model based on multimodal ultrasound.

In this study, two groups of statistically significant nodule ultrasonic features were selected as independent variables, and PTC was taken as dependent variable, so as to construct a PTC CHAID DT prediction model based on multimodal ultrasound technology, in which the ratio of parent-child nodes was set to 2:1. The results show that the root node of the model is E_max_. Through the test of the ten-fold cross-validation method, the prediction accuracy of CHAID DT model reached 82.4%, as shown in **Figure 3**.

### 3.5 Logistic regression model and DT model to predict PTC ROC curve drawing and analysis

**Figure 4:**
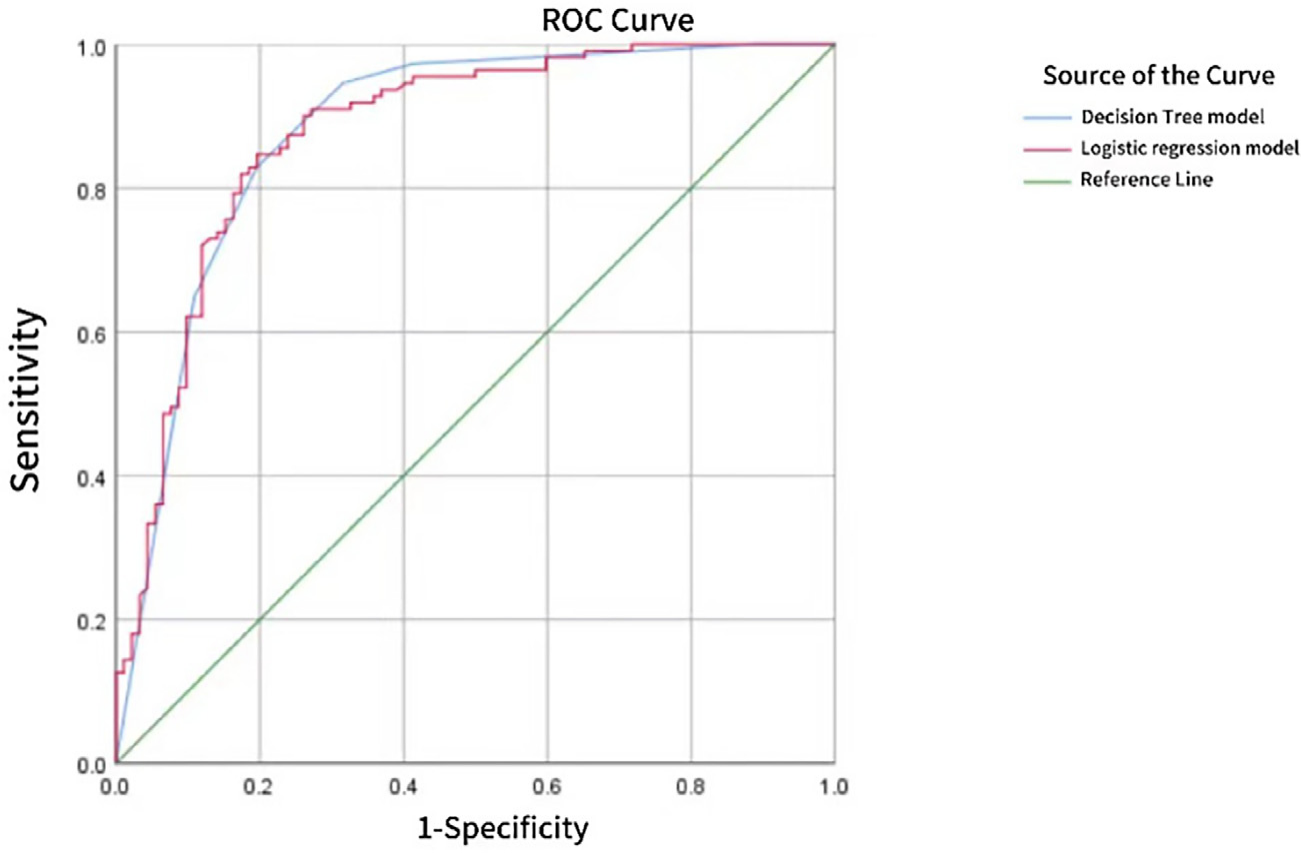
ROC curve of PTC predicted by Logistic regression model and CHAID DT model.

With pathological results as the gold standard, the binary Logistic regression model was used to predict the probability value of PTC, and the ROC curve was drawn. AUC was 0.878, standard error was 0.025, and 95%CI was 0.828~0.927 (P < 0.05). When 52.48% was used as the diagnostic limit value of prediction probability, the diagnostic sensitivity was 84.7%, the specificity was 80.4%, and the Yoden index was 0.651. The AUC of CHAID DT model was 0.883, the standard error was 0.025, 95%CI was 0.833~0.932 (P < 0.05), the diagnostic sensitivity was 82.9%, the specificity was 80.4%, and the Jorden index was 0.628, as shown in **Figure 4**. However, the difference between the two AUC was not statistically significant (z=0.325, P=0.7456).

## 4 Discussion

Although FNAB is the best minimally invasive method for the diagnosis of PTC, cytologic findings are useful in differentiating non-invasive follicular thyroid tumors with papillary nuclear features from the invasive peritumoral follicular variant of PTC (15). Scholars such as Sengul Ilker suggested that for butterfly gland, the thyroid should be 27-gauge finest needle in terms of US-FNA (16). But it also has some limitations, and the diagnosis is sometimes unsatisfactory. Multimodal ultrasound covers a variety of ultrasound techniques, including two-dimensional gray scale ultrasound, CDFI, ultrasound elastography, and CEUS. In recent years, with the gradual development of various ultrasound technologies, SWE and CEUS have been widely used in clinical practice. At the same time, with the continuous improvement of computer data mining technology, the ultrasonic examination results of patients with suspicious thyroid nodules are analyzed in a scientific, non-invasive, convenient and quick method, which has important help and important practical significance for the individualized and accurate clinical treatment.

In this study, 203 cases of thyroid nodules were collected and the Logistic regression model was established. It was concluded that thyroid nodules with low or very low echo, aspect ratio ≥1 and microcalcification under routine ultrasound were risk factors for PTC. Among 111 patients with PTC, 65.8% had an aspect ratio ≥1, which may be due to the rapid growth and diffusion of malignant nodules, which made the edges of nodules blurred, the lack of complete envelope, and the enlargement of the anterior-posterior diameter of nodules, which made the ultrasonography show that the aspect ratio was ≥1. However, some scholars have pointed out that as the size of the nodules increases, the value of the aspect ratio ≥1 in determining whether the nodules are benign or malignant gradually decreases (17). This study found that there was no significant correlation between the maximum diameter of nodules and the benign and malignant nodules. Demet Sengul et al. also found that nodule diameters of 10mm, 15mm and 20mm could not be used as cutoff values for predicting benign and malignant thyroid nodules with cytological uncertainty (18, 19). The growth of nodules may be limited by the thyroid envelope in both its anterior and posterior directions, which further leads to enhanced infiltration of nodules in the upper and lower or left and right directions. Calcium deposits can be present in different shapes, such as microcalcification (less than 2mm in diameter), coarse calcification, marginal calcification (eggshell-like calcification at the edge of nodules). This study showed that calcification existed in more than half of PTC, among which the number of PTC with strong echo point accounted for 50.5 (56/111), which was significantly higher than that of non-PTC group (22/92). This suggests that calcification is present not only in malignant nodules, but also in benign nodules. But microcalcification is seen as a sign of tissue degeneration and is often associated with cancer necrosis (20). Microcalcification is considered by many researchers to be an independent risk factor for malignant thyroid nodules, with a diagnostic specificity of up to 90% (21, 22). Its formation may be closely related to the rapid growth of follicular epithelial cells, newly formed vascular networks, mesenchymal cells, calcium salt accumulation and fibrotic processes. In addition, Peng et al. reported that a particular type of calcification, defined as “petal-like,” occurs more frequently in thyroid cancers with a higher incidence of lymph node metastasis (23). In this study, there were no obvious blood flow signals in the two groups of nodules, or only a few punctate blood flow signals were seen. The proportion of nodule with grade 0 and grade I blood flow signal in PTC group and non-PTC group was 90.1% and 85.8%, respectively, and there was no statistical difference in blood flow distribution in benign and malignant nodule (P=0.256). This is the same as Zhou et al. ‘s view that blood flow signals in PTC have no clear significance for the diagnosis of benign and malignant nodules (24). However, the study of Zhang Xia et al. is contrary to this study, they hold such a view: malignant tumors have the ability to generate angiogenic factors, which can activate the tumor and its surrounding tissues, thus generating new tumor blood vessels, which is why PTC usually shows rich blood flow signals (25). The reason for the difference may be insufficient sample size or differences between nodules. Since benign and malignant thyroid nodules overlap to a certain extent in conventional ultrasound images, we need to combine other ultrasound imaging techniques for identification.

This study found that the AUC of the ROC curve for diagnosing PTC in E_max_, E_min_ and E_mean_ were 0.842, 0.829 and 0.840, respectively, but there was no statistical significance among them (P > 0.05). In this study, E_max_ with relatively large AUC was selected for subsequent study. And this study found that the diagnostic critical value of E_max_ alone in diagnosing PTC was 41.25kPa. The CHAID DT model established in this study shows that the first layer is E_max_, indicating that the model believes that E_max_ has the strongest correlation with PTC. Xue et al. ‘s study also found that the best diagnostic value determined by ROC curve was E_max_ (26). However, some studies focusing on the differential diagnosis of SWE in benign and malignant thyroid nodules obtained different SWE parameters and different truncation values, and the truncation values even ranged from 25kPa to 90kPa (27). This may be due to the greater heterogeneity between nodules. In this study, the SWE parameters of PTC showed a high value, indicating that malignant nodules in the normal follicular cavity of the thyroid gland had a relatively high content of glia, while the content of connective tissue and blood vessels between the follicles and the interstitium was relatively rare. The cancerous tissue of PTC presents a papillary shape, with the formation of concentric calcium salt deposits in the substance, which is the growth of interstitial sand (psammoma body) and connective tissue, resulting in the formation of lymph nodules, which makes PTC harder than normal thyroid tissue. Microcalcification and coarse calcification of benign nodular clusters may also lead to a significant increase in hardness. However, Malhi H et al. concluded that scattered hyperechoic lesions in benign nodules were not risk factors for false positive results of SWE (28). It may be that some of the hyperechoic lesions scattered within benign nodules are not true calcifications, but concentrated colloid or fibrosis. Lei Zhu et al. found that the area under ROC curve of another elastic technology, namely real-time elastography (RTE), and combined FNA to distinguish benign and malignant thyroid nodules were 0.78 and 0.89, respectively (29, 30). However, SWE is more specific than RTE, and RTE is more dependent on physician techniques, which is easy to cause measurement bias (31, 32).

In this study, more than half of the PTC in CEUS examination was enhanced centripetal (56/111), which was consistent with the views proposed by Haugen BR et al. (33). This phenomenon may be caused by the uneven distribution of blood vessels in malignant nodules, which are divided into two parts: central blood supply and peripheral blood supply. The blood vessels in the middle are relatively few, while the blood vessels in the periphery are relatively dense. In this study, 66 of the malignant nodules were found to show low enhancement, which may be due to neovascular damage, poor function or dense fibrosis of tumor cells, and growth heterogeneity (34). Both Zhang and Jin et al. found that in thyroid nodules ≥1cm, no matter benign or malignant, most of CEUS did not show low enhancement, but equal-enhancement or high-enhancement (35, 36). This may be due to the accumulation of blood in the lesion during the growth of the nodules. In this study, there were also 45 cases (40.5%) of equal or high enhancement in non-PTC, which may be due to the abundant blood supply within the tumor or the high expression level of pro-angiogenesis factor promoted angiogenesis and tumor tissue differentiation, so that the perfusion volume in the tumor was similar to or relatively increased compared with normal thyroid tissue. In this study, the multi-factor analysis before the Logistic regression model was conducted found that CEUS characteristics did not become an independent risk factor for PTC (all P > 0.05), so there was no CEUS index in the model. However, in the CHAID DT model, whether the contrast agent is evenly distributed and the contrast agent infusion time become the nodes of the tree. This means that CEUS cannot be used alone for the diagnosis of thyroid cancer, but should be combined with conventional ultrasound and elastography. The CEUS technique is not yet fully standardized, there is no fixed reference for quantitative or qualitative evaluation, and no CEUS parameter has a high enough sensitivity and specificity to diagnose thyroid cancer (37).

Although ultrasound can distinguish benign and malignant nodules by specific imaging features, two-dimensional ultrasound cannot accurately determine “the same picture of different diseases”. For example, in the case of Hashimoto’s thyroiditis, thyroid tissue will be destroyed due to extensive lymphocyte infiltration and fibrosis, showing images very similar to malignant thyroid nodules, and it is easy to misdiagnose. Conventional CDFI uses wall filtering technology to eliminate clutter and motion artifacts, which results in tiny vessels in low-velocity blood flow that cannot be accurately detected, or because newly formed nodular vessels are not fully mature. The presence of blood flow signals alone cannot be the evidence for thyroid cancer, but the combination of resistance index and peak flow rate can assist in diagnosis. Therefore, further studies are needed to evaluate benign and malignant nodules by CDFI. The SWE imaging technique provides us with a color-coded map of the elastic values of the region, which helps us quantitatively determine the hardness of the tissue. However, SWE is also affected by the size and internal structure of the lesion. When the lesion is too large or too small or there is coarse calcification inside, the measurement of Young’s modulus will be affected, resulting in unsatisfactory measurement results. The formation of neovascularization and the growth of irregular vascular structures are prominent features of thyroid malignancies. Thyroid cancers smaller than 1cm are often characterized by low-intensity enhancement during CEUS testing. This is because the nodules are affected by the resolution of the instrument, and the nodules lack new blood vessels as well as abnormal blood vessels. However, some studies have shown that as nodules grow larger, their internal blood flow continues to increase, which makes thyroid malignant nodules with a diameter of 1-2cm usually show a trend of low to moderate enhancement, while thyroid cancers with a diameter of more than 2cm tend to show high enhancement (38, 39). Therefore, CEUS needs further study in differentiating benign and malignant thyroid nodules. Since each ultrasound technique has certain limitations, the combination of multimodal ultrasound may improve clinical diagnostic efficiency. Since each ultrasound technique has certain limitations, the combination of multimodal ultrasound may improve clinical diagnostic efficiency (14). Brandenstein et al. established a thyroid nodule scoring system using conventional ultrasound combined with shear wave elastography and CEUS, which showed good diagnostic efficacy in the detection of thyroid malignant nodules (40). In addition, a number of conventional ultrasound combined with elastography and multi-modal ultrasound techniques such as CEUS have shown good application prospects in the differential diagnosis of benign and malignant thyroid nodules, the diagnosis of nodules with uncertain cytological types, and the preoperative risk assessment of patients (41–43).

Statistical analysis in this study showed that the ACU of CHAID DT model and Logistic regression model to predict PTC based on multi-mode ultrasound was 0.883 and 0.878, respectively, but the difference was not statistically significant (z=0.352, P=0.7456). This shows that both models have a good diagnostic effect on PTC, but the diagnostic efficiency is similar. A significant advantage of Logistic regression model is that it can analyze the dependence between dependent variables (such as benign and malignant thyroid nodules) and independent variables (such as various ultrasonic features), which means that the influence of one variable can be analyzed under the premise of keeping other variables unchanged, but there is a deficiency in reflecting the interaction between independent variables. The DT model classifies data based on the probability of occurrence of various independent variables (i.e., various ultrasonic features). The model has the features of intuitive graphics and simple algorithm, and can effectively show the interaction between various ultrasonic features. However, DT may also have some disadvantages such as overfitting, underfitting, and easy to be affected by noisy data.

The limitations of this study are as follows: the sample size is limited, and large-scale sample studies are needed in the future to verify the research results; The perfusion parameters of nodule were not quantitatively analyzed in conjunction with the time-signal intensity curve of CEUS, which can be supplemented by future studies.

## 5 Conclusion

Both Logistic regression model and CHAID decision tree model can play a good role in the diagnosis of PTC based on multi-modal ultrasound, but the diagnostic efficiency of both models is comparable. The ultrasonic characteristics of hypoechoic or very hypoechoic, aspect ratio ≥1, microcalcification, high SWE value, uneven distribution of contrast agent and later perfusion time than surrounding tissue are important for the diagnosis of PTC. The high value of SWE has predictive value for PTC, and the Logistic regression model and CHAID decision tree model constructed together with other ultrasonic characteristics show high ability in diagnosis. CEUS cannot diagnose PTC alone and needs to be combined with conventional ultrasound and ultrasound elastography. In conclusion, these two models provide new insights and ideas for PTC diagnosis.

## Data Availability

The raw data supporting the conclusions of this article will be made available by the authors, without undue reservation.
